# Development of Laser Ionization Techniques for Evaluation of the Effect of Cancer Drugs Using Imaging Mass Spectrometry

**DOI:** 10.3390/ijms150711234

**Published:** 2014-06-25

**Authors:** Hiroki Kannen, Hisanao Hazama, Yasufumi Kaneda, Tatsuya Fujino, Kunio Awazu

**Affiliations:** 1 Graduate School of Engineering, Osaka University, 2-1 Yamadaoka, Suita, Osaka 565-0871, Japan; E-Mails: kannen-h@mb.see.eng.osaka-u.ac.jp (H.K.); awazu@see.eng.osaka-u.ac.jp (K.A.); 2 Graduate School of Medicine, Osaka University, 2-2 Yamadaoka, Suita, Osaka 565-0871, Japan; E-Mail: kaneday@gts.med.osaka-u.ac.jp; 3 Graduate School of Science and Engineering, Tokyo Metropolitan University, 1-1 Minamiosawa Hachioji, Tokyo 192-0397, Japan; E-Mail: fujino@tmu.ac.jp; 4 Graduate School of Frontier Biosciences, Osaka University, 1-3 Yamadaoka, Suita, Osaka 565-0871, Japan; 5 The Center for Advanced Medical Engineering and Informatics, Osaka University, 2-2 Yamadaoka, Suita, Osaka 565-0871, Japan

**Keywords:** imaging mass spectrometry, laser ionization, anticancer drug, drug resistance, zeolite matrix

## Abstract

Recently, combined therapy using chemotherapy and photodynamic therapy (PDT) has been proposed as a means of improving treatment outcomes. In order to evaluate the efficacy of combined therapy, it is necessary to determine the distribution of the anticancer drug and the photosensitizer. We investigated the use of imaging mass spectrometry (IMS) to simultaneously observe the distributions of an anticancer drug and photosensitizer administered to cancer cells. In particular, we sought to increase the sensitivity of detection of the anticancer drug docetaxel and the photosensitizer protoporphyrin IX (PpIX) by optimizing the ionization-assisting reagents. When we used a matrix consisting of equal weights of a zeolite (NaY5.6) and a conventional organic matrix (6-aza-2-thiothymine) in matrix-assisted laser desorption/ionization, the signal intensity of the sodium-adducted ion of docetaxel (administered at 100 μM) increased about 13-fold. Moreover, we detected docetaxel with the zeolite matrix using the droplet method, and detected PpIX by fluorescence and IMS with α-cyano-4-hydroxycinnamic acid (CHCA) using the spray method.

## 1. Introduction

In chemotherapy for cancer, the curative effect of an anticancer drug decreases with time because the cancer cells acquire drug resistance [1,2]. For example, docetaxel is often used to treat prostate cancers, but the response rate of docetaxel is not high (44.2% when used with prednisolone [3]), and the drug-resistant cells that arise are refractory to treatment. Consequently, new methods for treating drug-resistant tumors are urgently needed. One proposal for increasing the efficacy of treatment involves combining chemotherapy with photodynamic therapy (PDT). In PDT, the patient is treated with a photosensitizer that selectively accumulates in the tumor tissue, and then the tumor is targeted with a laser or other light source in order to excite the photosensitizer. However, current techniques for PDT do not exert a sufficient curative effect [4,5]. When chemotherapy and PDT are combined, the anticancer drug–resistant cells are killed by PDT, resulting in treatment that is more effective than PDT alone [6,7]. 

In order to evaluate the efficacy of combined therapy, it is important to determine the distributions of the anticancer drug and photosensitizer. Autoradiography is the standard means for determining drug distributions, but conventional drug imaging by autoradiography cannot be used to simultaneously observe multiple drugs [8]. Such simultaneous observation can be achieved using imaging mass spectrometry (IMS) of drugs, their metabolites, and other molecules in the biological tissue. However, the major disadvantage of the IMS is that its detection sensitivity is inferior to that of autoradiography. Moreover, a large difference in the ionization efficiencies of the drugs of interest can result in ion suppression (*i.e.*, obstruction of the ionization of the drug with lower ionization efficiency). 

To resolve this problem, we are investigating ways to use IMS to simultaneously observe the distributions of anticancer drug and photosensitizer administered to cancer cells. In IMS, matrix-assisted laser desorption/ionization (MALDI) is often used as the ionization method because of its sensitive solid sampling and soft ionization technique, which has been extensively applied to the analysis of macromolecules and low-molecular weight compounds [9,10,11,12,13]. The sensitivity of detection of a drug of interest by MALDI can be greatly enhanced by the use of ionization-assisting reagents. The purpose of this research was to increase the sensitivity of detection for the anticancer drug and photosensitizer administered to human prostate cancer cells, first by optimizing the ionization-assisting reagents used in the process, and then by investigating a new technique for simultaneous imaging of the anticancer drug and photosensitizer. 

## 2. Results and Discussion

### 2.1. Ionization of Docetaxel Using Zeolite Matrix

**Figure 1:**
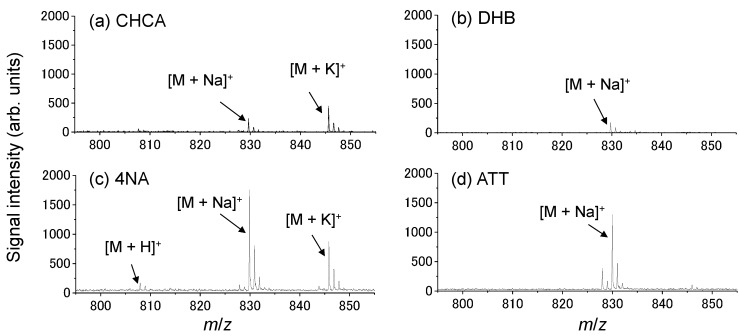
Typical mass spectra obtained from a the spot of the docetaxel using (**a**) α-cyano-4-hydroxycinnamic acid (CHCA); (**b**) 2,5-dihydroxy benzoic acid (DHB); (**c**) 4-nitroaniline (4NA); and (**d**) 6-aza-2-thiothymine (ATT). M indicates docetaxel.

**Figure 2:**
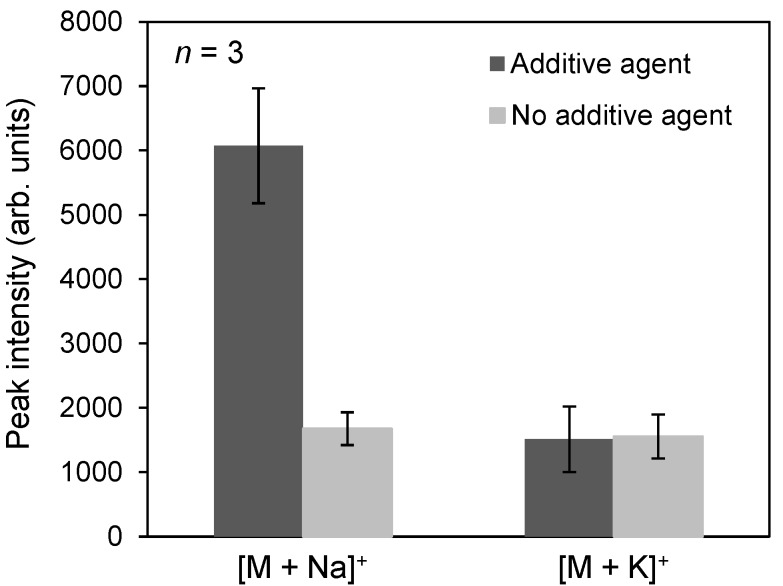
Relationship between the signal intensities of [M + Na]^+^ and [M + K]^+^. Black and gray bars mean that additive agent or no additive agent, respectively. M indicated docetaxel.

With the goal of enhancing the sensitivity of detection for anticancer drugs and photosensitizers, we attempted to optimize the ionization-assisting reagents used in the detection process. To this end, we investigated four conventional matrices: α-cyano-4-hydroxycinnamic acid (CHCA), 2,5-dihydroxy benzoic acid (DHB), 6-aza-2-thiothymine (ATT), and 4-nitroaniline (4NA). Figure 1 shows the mass spectra obtained from a 1-µL spot of 100 µM docetaxel using each matrix. Protonated, sodium-adducted, and potassium-adducted ions of docetaxel were detected. The peak of the sodium-adducted ion of docetaxel was most intense when 4NA was used as the matrix, but the ionization efficiency was not sufficient to allow detection of docetaxel administered to human prostate cancer cells (PC-3). To enhance the detection sensitivity further, we investigated the use of an additive dissolved in the matrix solvent, e.g., sodium acetate or potassium acetate, because the peak intensity of the sodium-adducted ion of docetaxel was the highest among the three peaks we detected. Figure 2 shows the average peak intensities of the sodium-adducted and potassium-adducted ions of docetaxel when sodium acetate or potassium acetate were added to the matrix solvent. Although the peak intensity increased about 4-fold, the ionization efficiency was still not sufficient to allow detection of docetaxel administered to PC-3 cells. 

**Figure 3:**
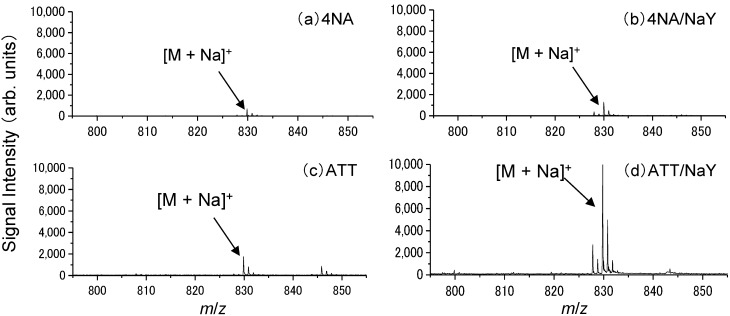
Typical mass spectra obtained from docetaxel using (**a**) 4NA; (**b**) 4NA/NaY; (**c**) ATT; (**d**) ATT/NaY with no additive agents. M indicates docetaxel.

**Figure 4:**
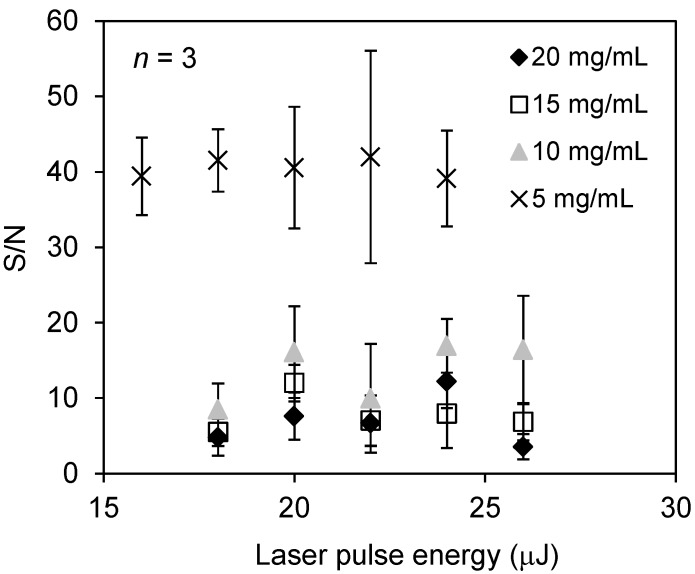
Relationship between S/N ratio of the docetaxel peak using zeolite matrix with no additive agents, at the indicated concentrations and laser pulse energies.

**Figure 5:**
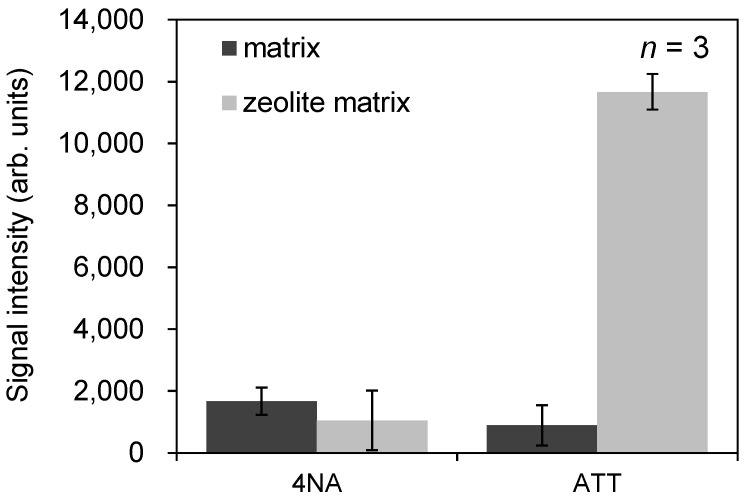
The average of peak intensity of the sodium-adducted ion of docetaxel using zeolite matrix with 4NA or ATT with no additive agents.

Next, in order to further enhance the detection sensitivity of docetaxel, we compared conventional matrix to a mixture of conventional matrix and zeolite matrix, a new ionization-assisting reagent that has been reported to increase the sensitivity of detection [14]. For this experiment, we selected NaY5.6 zeolite because it contains the sodium ion in its structure; as noted above, the peak intensity of sodium-adducted docetaxel was elevated when sodium acetate was added to the matrix solvent. Figure 3 shows typical mass spectra obtained from a spot of 100 μM docetaxel using the conventional matrix (ATT or 4NA) either alone or in combination with a zeolite matrix (NaY5.6). The peak intensity of the sodium-adducted ion [M + Na]^+^ was increased. Figure 4 shows the relationship between the signal-to-noise (S/N) ratio of the docetaxel peak in the presence of zeolite matrix at each concentration and laser pulse energy. Zeolite matrix prepared at 5 mg/mL was the most suitable for ionization of docetaxel on a metal sample plate; at a zeolite matrix concentration of 1 or 3 mg/mL, docetaxel could not be detected. Figure 5 shows the average peak intensity of the sodium-adducted ion of docetaxel with 4NA or ATT, in the presence or absence of zeolite matrix. When zeolite matrix was used with ATT, the detection sensitivity of the sodium-adducted docetaxel peak increased 13-fold. By contrast, when zeolite matrix was used with 4NA, the detection sensitivity was not increased. Using the Voyager DE-PRO TOF instrument, the detection limit of docetaxel using zeolite matrix was 1 μM. However, another MALDI-TOF instrument, the Ultraflex III, could obtain a peak at a lower concentration, 0.1 μM. Thus, docetaxel and PpIX in the PC-3 cells could easily be detected using the conventional MALDI matrix and a modern time of flight (TOF) instrument. 

### 2.2. Detection of Docetaxel Administered to PC-3 Cells

**Figure 6:**
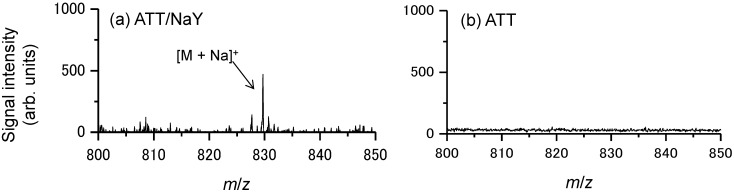
Typical mass spectra obtained from the prostate cancer cells (PC-3) cells administered 100 μM docetaxel using (**a**) the zeolite matrix; ATT/NaY or (**b**) the conventional matrix, ATT.

**Figure 7:**
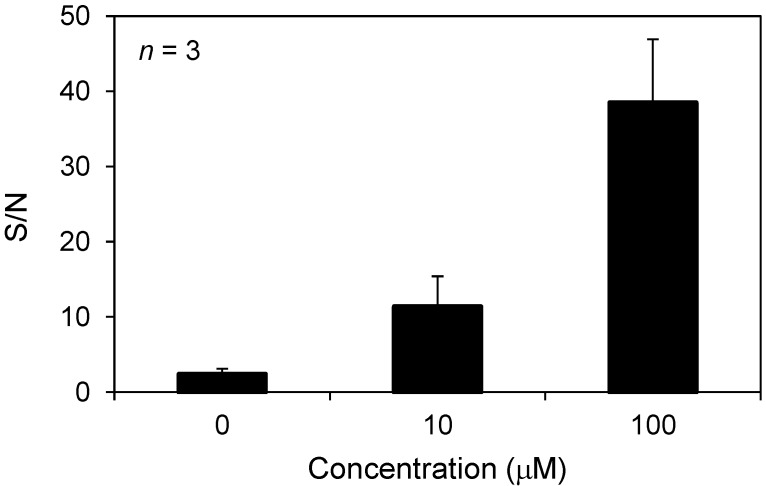
S/N ratio of the peak of [M + Na]^+^ peak at 0, 10 and 100 μM docetaxel.

As a first step toward acquiring images of docetaxel in prostate cancer tissue, we attempted to detect docetaxel administered to PC-3 cells using the zeolite matrix, ATT/NaY. Figure 6 shows typical mass spectra obtained from PC-3 cells administered 100 μM docetaxel using (a) zeolite matrix or (b) conventional matrix using the droplet method. Only in condition (a), the sodium-adducted ion of docetaxel [M + Na]^+^ was detected from a spot of PC-3 cells. Figure 7 shows the S/N ratio of the [M + Na]^+^ peak at concentrations of 0, 10, and 100 μM. [M + Na]^+^ was detected from PC-3 cells administered 10 μM docetaxel. In this method, we observed the “sweet spot” effect. Therefore, each mass spectrum used for analysis was the average of 10 mass spectra. 

### 2.3. Imaging of Cancer Drugs Administered to PC-3 Cells

**Figure 8:**
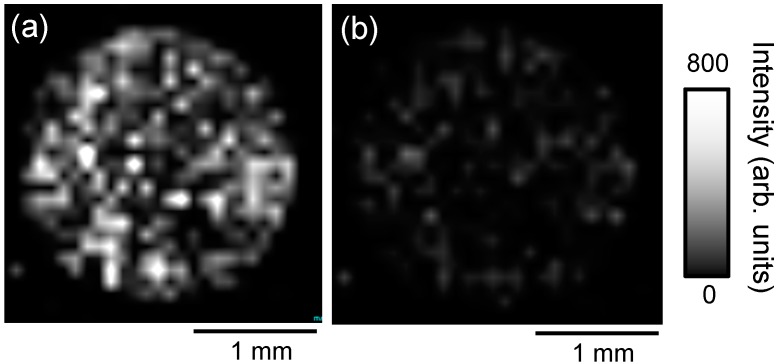
Ion image of (**a**) *m*/*z* 829.5 [D + Na]^+^; and (**b**) *m*/*z* 563.3 [P + H]^+^ obtained from PC-3 cells administered 50 μM docetaxel and 10 μM protoporphyrin IX (PpIX) using the zeolite matrix, ATT/NaY with the droplet method.

The use of the zeolite matrix enhanced the ionization efficiency of docetaxel sufficiently to allow detection of the drug in cancer cells. Next, we investigated ways to use IMS to simultaneously observe the distributions of anticancer drug and photosensitizer administered to cancer cells. First, we attempted IMS of docetaxel and PpIX administered to PC-3 with the zeolite matrix using the droplet method. Figure 8 shows the ion image of (a) *m*/*z* 829.5 (sodium-adducted docetaxel) and (b) *m*/*z* 563.1 (protonated PpIX) obtained from cultured PC-3 administered cells treated with 50 μM docetaxel and 10 μM PpIX, obtained with the zeolite matrix, ATT/NaY using the droplet method. In previous studies, the peak intensity of docetaxel was reduced when conventional matrices were used. In this study, however, docetaxel administered to cancer cells could be detected using zeolite matrix, whereas PpIX could not be detected in the same samples. 

### 2.4. Imaging of PpIX Administered to PC-3 Cells

**Figure 9:**
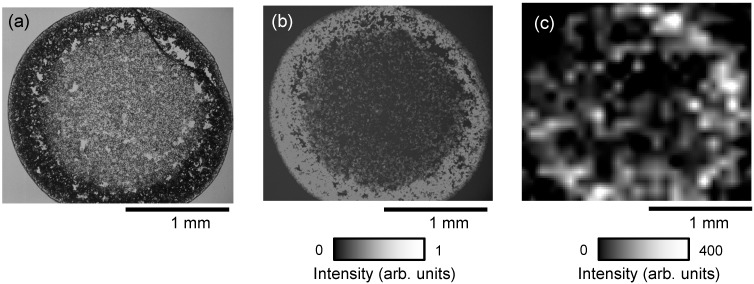
(**a**) Optical bright field image; (**b**) fluorescence image; and (**c**) ion image measured with CHCA using the spray method, obtained from the same PC-3 cells administered 10 μM PpIX.

Although docetaxel administered to PC-3 cells could be detected with the zeolite matrix, PpIX could not be detected by IMS in the same samples. To address this issue, we investigated a new combined method. Initially, we had acquired optical and fluorescence images of the PC-3 cells, and also detected docetaxel by IMS. The new method was developed to allow acquisition of the ion image by IMS after acquisition of the fluorescence and optical images. Figure 9 shows (a) optical bright field image, (b) fluorescence image, and (c) ion image measured with CHCA using the spray method. All images were obtained from the same cultured PC-3 cells administered 10 μM PpIX. Under these conditions, PpIX could be detected, and the distribution of PpIX in the ion image approximately corresponded to that in the fluorescence image. In MALDI imaging, docetaxel and PpIX could not be detected in the same sample. However, using the new method, it was possible to obtain both fluorescence images and IMS from the same cells. Thus, using this method, docetaxel and PpIX could be detected in the same sample, by MALDI imaging and fluorescence imaging, respectively. We will seek to confirm this in a future study with cellular scale using a stigmatic imaging mass spectrometer, MULTUM-IMG2 [15,16].

## 3. Experimental Section

### 3.1. Chemical and Reagents

Docetaxel is frequently used to treat prostate cancer. Docetaxel trihydrate was purchased from Wako (Osaka, Japan). PpIX was purchased from SIGMA-Aldrich (Tokyo, Japan). CHCA, DHB, 4NA, and ATT, used as the matrix, were purchased from Sigma-Aldrich (Tokyo, Japan). Acetonitrile and methanol (SIGMA-Aldrich, Tokyo, Japan) were used as the matrix solvent. The zeolite, NaY5.6, was supplied by the Catalysis Society of Japan (Tokyo, Japan).

### 3.2. Cell Culture

The cell line used was the hormone-antagonistic human prostate cancer cell line PC-3. Cells were cultured in Dulbecco’s modified Eagle’s medium (D-MEM, D6046, SIGMA-Aldrich, Tokyo, Japan) containing 10% fetal bovine serum (FBS, S1820, Biowest SAS, Nuaillé, France) and 100 units/mL each of penicillin and streptomycin (T4049, SIGMA-Aldrich, Tokyo, Japan). Cells were incubated at 37 °C in an atmosphere containing 5% CO_2_.

### 3.3. Mass Spectrometer

All experiments were carried out in positive mode with reflectron-mode using Voyager DE-PRO TOF mass spectrometer (Applied Biosystems, Foster City, CA, USA) equipped with a 355-nm third-harmonic Nd:YAG laser (GAIA II 30-T, Rayture Systems Co., Ltd., Tokyo, Japan). Instrument parameters on the reflectron-mode of Voyager DE-PRO were as follows: accelerating voltage, +20 kV; voltage for the extraction grid, +13.6 kV; voltage for the guide wire, 0 V; extraction delay time, 100 ns.

### 3.4. Sample Preparation

For the conventional matrices, CHCA was dissolved in 1:1 (*v*/*v*) acetonitrile:water (*v*/*v*) at 10 mg/mL, and DHB, 4NA, and ATT were dissolved in 1:1 (*v*/*v*) methanol:water at 30, 5, and 10 mg/mL, respectively. Sodium acetate or potassium acetate was used as an additive agent and dissolved in the matrix solvent at 10 mM. For the zeolite matrices, 4NA or ATT was mixed with an equal weight of NaY5.6 zeolite with a mortar for over 10 min, and then suspended in 1:1 (*v*/*v*) methanol:water. To optimize the matrix for ionization of docetaxel, initially a 1-μL drop of docetaxel was spotted onto a metal sample plate, and then dried under vacuum; the each matrix was placed on dried spot of docetaxel. In this experiment, either indium tin oxide (ITO)-coated glass slide (# 237001, Bruker, Billerica, MA, USA) or a metal plate (4347686, Applied Biosystems, Foster City, CA, USA) was used as the sample plate. From experiments described in Section 2.1, Section 2.2 and Section 2.3, the metal sample plate was used. For the experiment described in Section 2.4, the ITO-coated glass slide was used to acquire the optical images. To compensate for the low conductivity of the glass slide, which caused a charge increase relative to the metal sample plate, the ITO glass slide was coated with gold at a thickness of 10 nm using an ion-sputtering device (E-1010, HITACHI, Tokyo, Japan). Then, PC-3 cells were spotted onto the ITO glass slide, and an optical bright field image and fluorescence image were acquired after the spot was dried under vacuum. Before matrix application, Gold was again coated at a thickness of 10 nm. Matrix was applied using an airbrush (PS-153: 3500 PRO SPRY Mk-2, Mr. hobby, GSI Creos Co., Tokyo, Japan). Because it was possible that the metal nozzle would be degraded by the acidity of the matrix, reducing the intensities of the signals of interest, the nozzle was replaced with a microchip (DIAMOND TIP 0.1–10 μL, F171100, Gilson, Middleton, WI, USA) and a glass tube (Calibrated Pipets, 2-000-001-90, Drummond Scientific Co., Broomall, PA, USA).

### 3.5. Drug Administrations into Cells

Docetaxel and PpIX were dissolved in dimethyl sulfoxide (DMSO, D4540, SIGMA-Aldrich, Tokyo, Japan). The solution of docetaxel or PpIX was diluted 100-fold in medium containing 10% fetal bovine serum or not, respectively. PC-3 cells were incubated for 3 h in the medium containing each drug. After each drug administration, the cells were washed once with phosphate-buffered saline (PBS, D8537, SIGMA-Aldrich, Tokyo, Japan), trypsinized, resuspended in PBS, and centrifuged. After removal of the supernatant, a suspension of PC-3 cells at 1.0 × 10^7^ cells/mL was prepared by addition of distilled water.

### 3.6. Microscopy

The optical bright field and fluorescence images of PpIX administered to PC-3 cells were acquired using an inverted fluorescence microscope (LEITZ DMIRB, Leica Microscope and System GmbH, Wetzlar, Germany) equipped with a mercury lamp (02651000, IREM, Borgone Susa, Italy) as the excitation light source. Exposure time was set at 20 s.

## 4. Conclusions

In order to evaluate the efficacy of combined therapy, it is important to determine the distributions of the anticancer drug and photosensitizer. The purpose of this research was to enhance the sensitivities of detection of anticancer drug and photosensitizer administered to PC-3 human prostate cancer cells by improving the ionization assisting reagents used in the process. When we used a matrix consisting of equal weights of zeolite (NaY5.6) and the conventional organic matrix 6-aza-2-thiothymine (ATT) in matrix-assisted laser desorption/ionization, the signal intensity of the sodium-adducted ion of docetaxel (administered at 100 μM) increased about 13-fold. Moreover, docetaxel administered to PC-3 cells could be detected with zeolite matrix using the droplet method. Although the ion image of docetaxel and PpIX could not be acquired simultaneously, PpIX administered to PC-3 cells could be detected in a fluorescence image, after which we could successfully detect PpIX in the same samples by IMS with CHCA using the spray method. These results demonstrate that it is possible to detect docetaxel in IMS using a zeolite matrix, following the detection of PpIX by fluorescence imaging. 

## References

[1] Kaufmann S., Vaux D. (2003). Alterations in the apoptotic machinery and their potential role in anticancer drug resistance. Oncogene.

[2] Patterson S.G., WEI S., Chen X., Sallman D.A., Gilvary D.L., Zhong B., Pow-Sang J., Yeatman T., Djeu J.Y. (2006). Novel role of Stat1 in development of docetaxel resistance in prostate tumor cells. Oncogene.

[3] The Interview Form of Taxotere^®^ (Docetaxel for Injec-tion, Sanofi-Aventis). http://www.sec.gov/Archives/edgar/data/1121404/000119312511050947/d20f.htm.

[4] Thompson M.S., Jonansson T., Andersson-Engels S., Svanberg S., Bendsoe N., Svanberg K. (2005). Clinical system for interstitial photodynamic therapy with combined on-line dosimetry measurements. Appl. Opt..

[5] Yamauchi M., Honda N., Hazama H., Tachikawa S., Nakamura H., Kaneda Y., Awazu K. (2014). A novel photodynamic therapy for drug-resistant prostate cancer cells using porphyrus envelope as a novel photosensitizer. Photodiagn. Photodyn. Ther..

[6] Zuluaga M.F. (2008). Combination of photodynamic therapy with anti-cancer agents. Curr. Med. Chem..

[7] Sinha A., Anand S., Ortel B., Chang Y., Mai Z., Hasan T., Maytin E. (2006). Methotrexate used in combination with aminolaevulinic acid for photodynamic killing of prostate cancer cells. Br. J. Cancer.

[8] Hsieh Y., Casale R., Fukuda E., Chen J., Knemeyer I., Wingate J., Morrison R., Korfmacher W. (2006). Matrix-assisted laser desorption/ionization imaging mass spectrometry for direct measurement of clozapine in rat brain tissue. Rapid Commun. Mass Spectrom..

[9] Russell D.H., Edmondson R.D. (1997). High-resolution mass spectrometry and accurate mass measurements with emphasis on the characterization of peptides and proteins by matrix-assisted laser desorption/ionization time-of-flight mass spectrometry. J. Mass Spectrom..

[10] Gusev A.I., Wilkinson W.R., Proctor A., Hercules D.M. (1995). Improvement of signal reproducibility and matrix/comatrix effects in MALDI analysis. Anal. Chem..

[11] Billeci T.M., Stults J.T. (1993). Tryptic mapping of recombinant proteins by matrix-assisted laser desorption/ionization mass spectrometry. Anal. Chem..

[12] Cohen S.L., Chait B.T. (1996). Influence of matrix solution conditions on the MALDI-MS analysis of peptides and proteins. Anal. Chem..

[13] Duncan M.W., Matanovic G., Cerpa-Poljak A. (1993). Quantitative analysis of low molecular weight compounds of biological interest by matrix-assisted laser desorption ionization. Rapid Commun. Mass Spectrom..

[14] Komori Y., Shima H., Fujino T., Kondo J.N., Hashimoto K., Korenaga T. (2010). Pronounced selectivity in matrix-assisted laser desorption-ionization mass spectrometry with 2,4,6-trihydroxyacetophenone on a zeolite surface: Intensity enhancement of protonated peptides and suppression of matrix-related ions. J. Phys. Chem..

[15] Hazama H., Aoki J., Nagao H., Suzuki R., Tashima T., Fujii K., Masuda K., Awazu K., Toyoda M., Naito Y. (2008). Construction of a novel stigmatic MALDI imaging mass spectrometer. Appl. Surf. Sci..

[16] Aoki J., Ikeda S., Toyoda M. (2014). Observation of accumulated metal cation distribution in fish by novel stigmatic imaging time-of-flight mass spectrometer. J. Phys. Soc. Jpn..

